# Discovery of a Novel Polymer for Human Pluripotent Stem Cell Expansion and Multilineage Differentiation

**DOI:** 10.1002/adma.201501351

**Published:** 2015-06-01

**Authors:** Adam D. Celiz, James G. W. Smith, Asha K. Patel, Andrew L. Hook, Divya Rajamohan, Vinoj T. George, Luke Flatt, Minal J. Patel, Vidana C. Epa, Taranjit Singh, Robert Langer, Daniel G. Anderson, Nicholas D. Allen, David C. Hay, David A. Winkler, David A. Barrett, Martyn C. Davies, Lorraine E. Young, Chris Denning, Morgan R. Alexander

**Affiliations:** ^1^ Laboratory of Biophysics and Surface AnalysisSchool of PharmacyUniversity of NottinghamNottinghamNG7 2RDUK; ^2^ Wyss Institute for Biologically Inspired Engineering at Harvard UniversityBostonMA02115USA; ^3^ Wolfson Centre for Stem CellsTissue Engineering and ModellingSchool of MedicineCentre for Biomolecular SciencesUniversity of NottinghamNottinghamNG7 2RDUK; ^4^ David H. Koch Institute for Integrative Cancer ResearchDepartment of Chemical EngineeringInstitute for Medical Engineering and ScienceMassachusetts Institute of TechnologyCambridgeMA02139USA; ^5^ CSIRO Manufacturing Flagship343 Royal ParadeParkville3052Australia; ^6^ Cardiff School of BiosciencesThe Sir Martin Evans BuildingMuseum AvenueCardiffCF10 3AXUK; ^7^ MRC Centre for Regenerative Medicine SCRM BuildingThe University of EdinburghEdinburgh BioQuarter, 5 Little France DriveEdinburghEH16 4UUUK; ^8^ CSIRO Manufacturing FlagshipBayview AvenueClayton3168Australia; ^9^ Monash Institute of Pharmaceutical Sciences399 Royal ParadeParkville3052Australia; ^10^ Latrobe Institute for Molecular ScienceLatrobe UniversityBundoora3086Australia; ^11^ Centre for Analytical BioscienceSchool of PharmacyUniversity of NottinghamNottinghamNG7 2RDUK

**Keywords:** high throughput, human pluripotent stem cells, materials discovery, polymer microarrays, stem cell differentiation

## Abstract

**A scalable and cost‐effective synthetic polymer substrate** that supports robust expansion and subsequent multilineage differentiation of human pluripotent stem cells (hPSCs) with defined commercial media is presented. This substrate can be applied to common cultureware and used off‐the‐shelf after long‐term storage. Expansion and differentiation of hPSCs are performed entirely on the polymeric surface, enabling the clinical potential of hPSC‐derived cells to be realized.

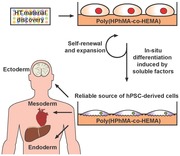

Human pluripotent stem cells (hPSCs) are proving valuable for various biomedical applications due to their ability to self‐renew and be differentiated into numerous lineages representative of the three embryonic germ layers.^1^, ^2^, ^3^, ^4^ We apply a high throughput materials discovery approach to identify a novel polymer for hPSC culture using microarray screening of an unprecedented chemical space (141 monomers, polymerized alone and mixed to form 909 unique polymers, tested in 4356 individual assays). This identified the first synthetic polymeric substrate that achieves both pluripotent hPSC expansion (in the commercially available culture media, StemPro and mTeSR1) and subsequent multilineage differentiation into representatives of the three germ layers, namely cardiomyocytes, hepatocyte‐like cells, and neural progenitors. A simple procedure was developed to coat standard cultureware with this polymer and notably, there was no need for protein preconditioning prior to use, which is a significant advance on previous polymers discovered by high throughput screening.^5^, ^6^, ^7^, ^8^

For the significant therapeutic potential of hPSCs to be realized, bioprocessing‐scale culture systems are required that can manufacture clinically relevant numbers of cells in an economical and reproducible manner. For adherent cell types, the ideal culture system should comprise both a defined culture medium and a substrate that can be readily used with existing cultureware.

Commercially available defined media formulations have improved reproducibility of hPSC expansion by avoiding mouse embryonic fibroblast‐conditioned medium, which remains commonplace but exhibits high batch variability.^9^, ^10^, ^11^ Nonetheless, there is still widespread use of the poorly defined mouse sarcoma preparation, Matrigel, as a cell attachment surface.^12^ Such xenogenic components create a barrier to clinical translation as they face greater regulatory hurdles. Progress has been made with polymeric materials that can be easily manufactured from inexpensive, readily available monomers and are readily scalable industrially.^13^ High throughput screening of polymer microarrays has identified polymers as able to support the clonal growth of human embryonic stem cells (hESCs).^5^ However, synthetic polymers identified by screening to date require preconditioning with a protein for cell attachment and pluripotent cell expansion, limiting their clinical and commercial applicability. Furthermore, both pluripotent expansion of hPSCs and multilineage differentiation has not been demonstrated on a single synthetic surface.

A multigeneration high throughput polymer microarray screening methodology incorporating high throughput surface characterization (HT‐SC) was used to identify materials that can support the attachment and pluripotency of the HUES7 hESC line in the widely used commercial defined, serum‐ and feeder‐free medium, StemPro.^14^ The first‐generation array, consisting of 141 monomers of wide chemical diversity (utilizing more than 90% of photo curable monomers that are readily commercially available), was printed using metal pins to transfer the liquid monomers onto poly(2‐hydroxyethyl methacrylate) (polyHEMA) coated glass slides as spots with six replicates of each homopolymer (**Figure** **1**a – monomer structures presented in Figure S1 in the Supporting Information).^15^ Polymer microarray spots of diameters ranging from 250 to 400 μm were formed by UV initiated photopolymerization using a modification of methods described previously which reduced spreading of the large library of monomers on the polyHEMA substrate prior to UV irradiation (Figure S2, Supporting Information).^16^, ^17^ Arrays were preconditioned for 1 h in StemPro medium prior to seeding with 1 × 10^6^ HUES7 hESCs and culturing for 24 h. Samples were fixed, stained for OCT4 expression (an indicator of pluripotency) and images acquired using an automated fluorescence microscope. Images were automatically processed to quantify cell response to each polymer spot (using CellProfiler software). This initial screen was used to identify 24 “hit” materials on the basis of their ability to support high HUES7 hESC attachment across six replicates whilst maintaining OCT4 expression (>90%) (Figure 1b,c – “hit” monomer structures presented in Figure S3 in the Supporting Information).

**Figure 1:**
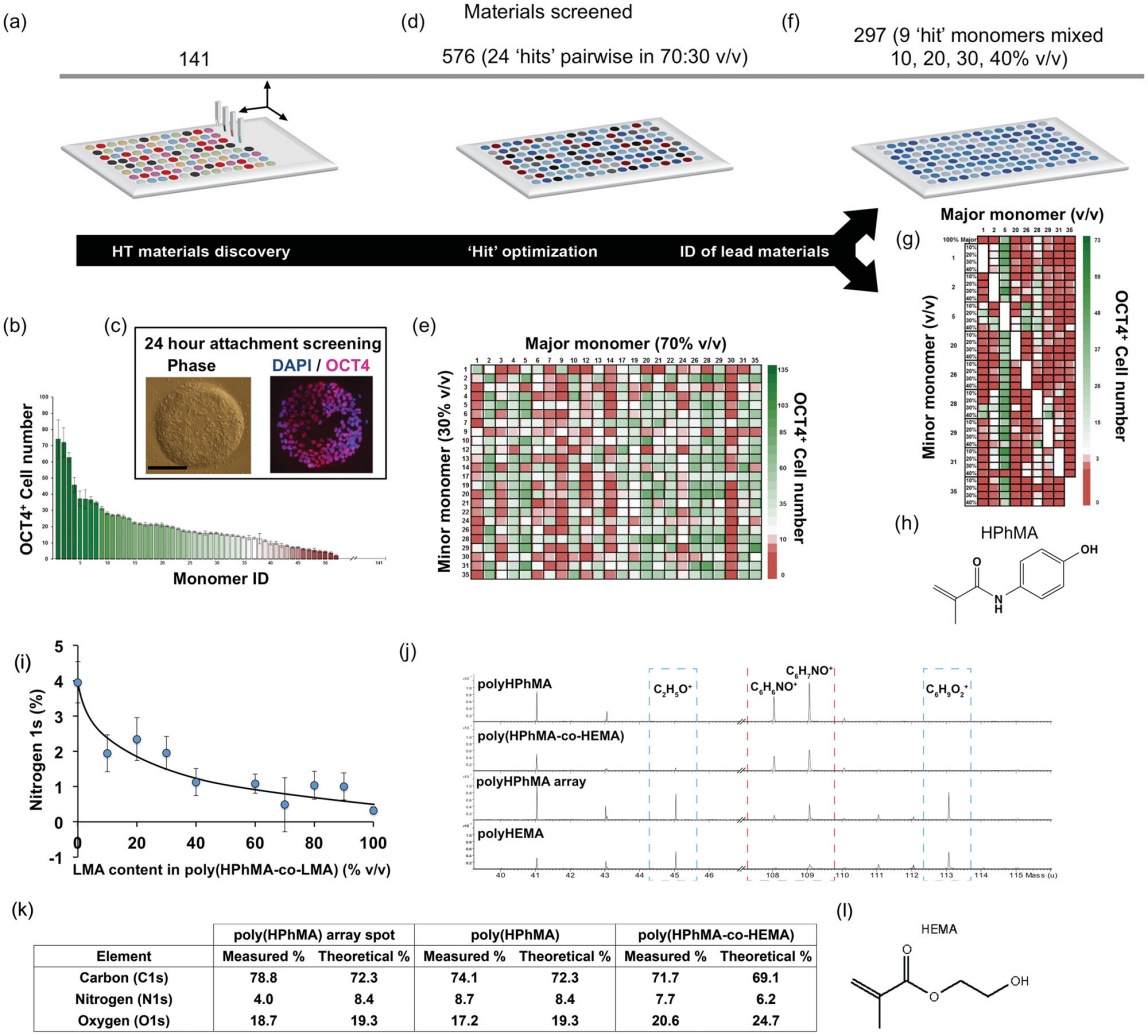
Multigeneration microarray screening strategy and HT‐SC. a) A first‐generation array of wide chemically diversity (utilizing more than 90% of photo curable monomers that are readily commercially available) was screened for hPSC attachment. b) Materials were ranked by OCT4^+^ hPSC attachment (six replicates) after 24 h in culture whereupon cells were quantified by DAPI and OCT‐4 staining c). d) Twenty‐four “hit” materials were mixed pairwise (70/30% v/v) in a combinatorial manner to produce a second‐generation of 576 unique materials which were assessed and ranked for OCT4^+^ hPSC attachment (e). f) Nine monomers were identified that formed various hit copolymers in the second‐generation array, these formed the third‐generation array but were mixed in further ratios to form an array of 297 materials which were ranked by OCT4^+^ hPSC attachment to identify lead compositions for scale up (g). h) Chemical structure of the HPhMA monomer. i) XPS analysis of polyHPhMA, polyLMA, and copolymers thereof to determine the actual surface chemistry. Line is drawn to guide the eye. j,k) ToF‐SIMS (j) and XPS (k) analysis of polyHPhMA in the third‐generation array revealed polyHEMA at the surface. l) Chemical structure of the HEMA monomer.

To explore the effect of copolymerization the 24 hit monomers were mixed pairwise (70/30% v/v mixtures to explore each monomer as a major and minor component of each copolymer) to form a second‐generation array design comprising 576 unique materials in triplicate (Figure 1d). Quantifying OCT4^+^ HUES7 cell attachment after 24 h on the second‐generation array in the same way as before identified a refined list of nine monomers that displayed high hPSC attachment as homopolymers and copolymers across the array (up to 100 cells spot^−1^) (Figure 1e). Synergistic combinations of monomers were noted, whereby greater hPSC attachment was observed for a copolymer than their homopolymer counterparts. However, no clear copolymer candidate could be identified to take forward for scale up purposes for hPSC expansion. A third‐generation array was used to explore hit monomers as copolymers at varied composition ratios to determine whether substrates could be improved further for HUES7 cell attachment and maintenance of OCT4 expression during the first 24 h of culture. Of the 24 monomers that were employed in the second‐generation array, nine monomers were taken forward to the third‐generation array as there were able to support high hPSC attachment as homopolymers and multiple copolymer formulations. The nine lead monomers were mixed combinatorially, utilizing additional ratios (10, 20, 30, and 40% v/v) to produce a third‐generation array of 297 materials (Figure 1f). To make the assay more stringent in order to identify the most robust candidate polymers, the cell seeding density was reduced. This led to a significant reduction in cell attachment across the array, with only 90 copolymers showing significant cell adhesion.

The best performing polymers in the third‐generation array all contained monomer 5 (*N*‐(4‐hydroxyphenyl)methacrylamide) (HPhMA), which was able to support HUES7 cell adhesion both as a homopolymer and as a copolymer (up to 56 ± 7 cells spot^−1^) (Figure 1g). Inclusion of HPhMA as a minor component (10%–40% v/v) with monomers that performed poorly as homopolymers dramatically increased the performance of the resulting copolymers. For example, monomer 26 (lauryl methacrylate) (LMA) supported no attachment of hPSCs across the array as a homopolymer. However, inclusion of HPhMA as a minor (10% v/v) or major (90% v/v) component with LMA increased hPSC attachment to 41 ± 15 and 38 ± 9 cells spot^−1^, respectively (Figure 1g). To investigate the excellent cell attachment performance of HPhMA‐containing copolymers in the third‐generation array, the intensity of the time‐of‐flight secondary‐ion mass spectrometry (ToF‐SIMS) ions characteristic to HPhMA (C_7_H_4_NO_2_^−^) and LMA (C_9_H_11_O_2_^+^) were compared in the spectra of the homopolymers and copolymers using high throughput surface characterization (Figure S4, Supporting Information). The intensity of the characteristic C_7_H_4_NO_2_^−^ secondary ion was highest in the polyHPhMA homopolymer and decreased dramatically upon inclusion of LMA. This can be explained by the surface enrichment of LMA, possibly in the monomer mixture prior to UV photopolymerization. The intensity of C_9_H_11_O_2_^+^ secondary ion characteristic of LMA was consistent with this explanation. To quantify the amount of HPhMA at the surface of these materials, X‐ray photoelectron spectroscopy (XPS) analysis was employed using the elemental abundance of nitrogen as a marker for polyHPhMA (Figure 1i). The relative amount of nitrogen in polyHPhMA homopolymer ([*N*] = 4 at%) was reduced by half upon inclusion of 10% LMA (1.9%). The amount of nitrogen in the XPS spectra follows a similar trend to the C_7_H_4_NO_2_^−^ ion in the ToF‐SIMS spectra for these materials confirming that LMA was enriched at the surface. Despite there being relatively lower levels of polyHPhMA at the surface of all copolymers than the uniform distribution expected of statistical copolymers high cell attachment was achieved on these polymers, suggesting that only small amounts of HPhMA are required to encourage cell attachment. Moreover, copolymer formulations did not significantly increase cell attachment over polyHPhMA. Therefore, our detailed analysis showed that the benefits of using a copolymer were modest on hPSC attachment and did not outweigh the added complexity of fabrication, analysis, and quality control relative to using a homopoly­mer. This meant we elected to take forward polyHPhMA for hPSC expansion studies.

Scaling up of polyHPhMA into six‐well plates was achieved (see methods) and analyzed by ToF‐SIMS to determine if the surface chemistry was consistent with polyHPhMA in microarray spots. ToF‐SIMS peaks characteristic of HPhMA were observed at *m*/*z* = 108 and 109 (C_6_H_6_NO^+^ and C_6_H_7_O^+^, respectively) from both microarray spots and from coatings scaled up to coat six‐well plates (Figure 1j). Although the polyHEMA substrate was used throughout the array screening process, we observed additional peaks at *m*/*z* = 45 and 113 (C_2_H_5_O^+^ and C_6_H_9_O_2_^+^, respectively) only in the third‐generation polymer microarray (likely due to thinner spots than previous generation arrays), which are characteristic of the polyHEMA slide coating indicating it had intermixed with the deposited monomers and was present at the surface of the spots of this array.

Transparent coatings of polyHPhMA were achieved by presynthesizing the polymer and dissolving in ethanol before casting onto plasma etched tissue culture polystyrene (PE‐TCPS) cultureware. Cracking within the coating of polyHPhMA was observed upon storage in cell culture incubators. The serendipitous discovery of the beneficial role of polyHEMA within polyHPhMA in the micro array format was utilized in the scaled up well plate experiments by conventional copolymerization of the two monomers (Figure 1h,l). Poly(HPhMA‐*co*‐HEMA) gave transparent coatings that did not crack, even after 1 month of incubation in medium. XPS analysis of poly(HPhMA‐*co*‐HEMA) coatings confirmed the composition of the material (Figure 1k).

To evaluate hPSC expansion on these substrates we first assessed whether preconditioning with culture medium or ECM proteins was required for hPSCs to retain pluripotency in six‐well plates coated with poly(HPhMA‐*co*‐HEMA). Attachment and distribution of hPSCs 24 h after seeding in StemPro was similar irrespective of preconditioning, as was the time required to reach confluency (72 h). Therefore, preconditioning was omitted in subsequent experiments. This approach enabled cells to be cultured through five serial passages with accutase on the poly(HPhMA‐*co*‐HEMA) substrate maintaining expression of OCT4, TRA181, and SSEA4 in >93% cells, as measured by quantitative immunofluorescence using an automated plate reader (Operetta) and high‐content image analysis software (CellProfiler), with retention of a 46,XY karyotype by G‐banding 30 cells (**Figures** **2**a,b and S5, Supporting Information).

**Figure 2:**
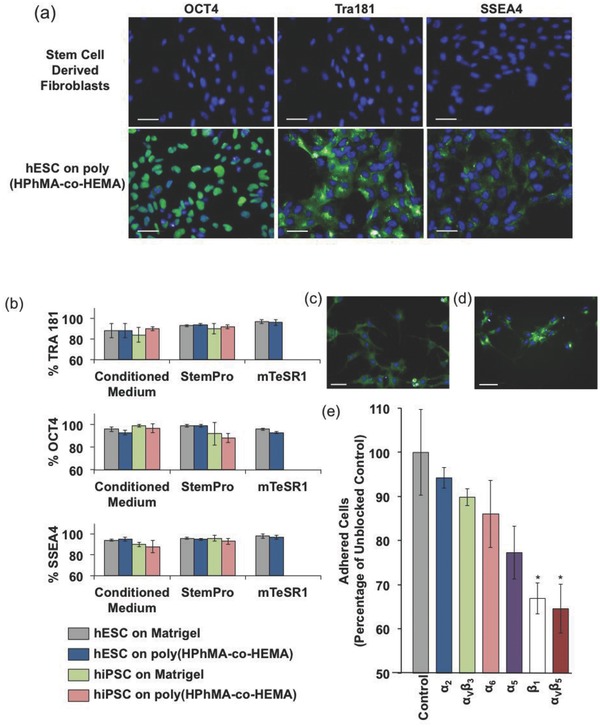
hPSC expansion through serial passage. a) Positive immunofluorescence for pluripotent markers OCT4, TRA181, and SSEA4 following serial passaging of hPSCs on poly(HPhMA‐*co*‐HEMA). Scale bar = 50 μm. b) hPSCs on poly(HPhMA‐*co*‐HEMA) maintain pluripotent marker expression levels, with OCT4, TRA181, and SSEA4 expression >88%. hESCs adhered to poly(HPhMA‐*co*‐HEMA) actively express the integrins c) β_1_ and d) α_V_β_5_. Scale bar = 50 μm. e) Blocking of integrins β_1_ and α_V_β_5_ significantly reduced hPSC adhesion to poly(HPhMA‐*co*‐HEMA) in StemPro media.

We next sought to explore whether the poly(HPhMA‐*co*‐HEMA) substrate could support pluripotent expansion of hESC and hiPSC lines in different commercial culture media. Thus, cultures of the HUES7 hESC line and BT1 hiPSC line were initiated in StemPro and another commonly used defined medium, mTeSR1.^9^ In each case the conditions supported proliferation through five serial passages, whilst retaining stable karyotype (46,XY for HUES7; 46,XX for BT1) (Figure S5, Supporting Information), and pluripotent marker expression of OCT4, TRA181, and SSEA4 by immunofluorescence (all >88%) (Figure 2a,b). Repeat experiments conducted on poly(HPhMA‐*co*‐HEMA)‐coated cultureware stored for at least 6 months at ambient conditions produced identical results, demonstrating that these coatings can be used off‐the‐shelf in the same way as general TCPS cultureware.

To determine a mechanism for the hPSC adhesion to poly(HPhMA‐*co*‐HEMA), antibody blocking assays were performed for key hPSC integrins. Blocking of the integrins β_1_ and α_V_β_5_ resulted in a significant reduction (>30%) in hPSC attachment to poly(HPhMA‐*co*‐HEMA) when cultured in StemPro media (Figure 2c–e). Although hPSCs have been shown to express numerous integrins, including those of the α_1_, α_2_, α_3_, α_5_, α_6_, α_7_, α_V_, and α_11_, and β_1_, β_2_, β_3_, and β_5_ families, only α_2_, α_5_, α_6_, α_V_, and β_1_ integrins have been shown to play a significant role in hPSCs adhesion to Matrigel coated culture surfaces and only α_V_ integrins in hPSC adhesion to polymer culture surfaces without matrix coatings.^5^, ^12^, ^18^, ^19^, ^20^, ^21^ This is therefore the first report demonstrating a role for β_1_ as well as α_V_ integrins in hPSCs adhesion to polymer culture surfaces without matrix coatings. Although individually α_V_β_5_ binds vitronectin sites and β_1_ binds fibronectin and laminin sites, it is likely that these two integrins interact in a complex manner to promote hPSC adhesion to sites present in the poly(HPhMA‐*co*‐HEMA) chemistry or to proteins adsorbed from the medium.^22^

Differentiation capacity would greatly increase the utility of expansion culture substrates. We, therefore, sought to evaluate whether the formation of representatives of each of the three germ layers during human development could be induced by directing differentiation on poly(HPhMA‐*co*‐HEMA).

We directed formation of cardiomyocytes (mesoderm) by culturing 2D monolayers of hPSCs on poly(HPhMA‐*co*‐HEMA) with modulators of the transforming growth factor beta (TGF‐β) superfamily (activin A and BMP4) and WNT (KY02111 and XAV393) pathways.^23^ In the same time course as hPSCs differentiated on Matrigel (12 d), beating clusters of cardiomyocytes spontaneously formed (see Video S1 in the Supporting Information), which were shown by immunostaining to be positive for α‐actinin and cardiac troponin‐T staining (**Figure** **3**a). Functional analysis of the differentiated cells by patch clamp showed they had electrophysiological characteristics similar to those previously published for hPSC‐cardiomyocytes, including a mean action potential duration (APD) of 417 + 102 ms (Figure 3b).^24^ Based on 90%/50% repolarization values (APD90/APD50), these cultures contained ventricular (APD90/APD50 of ≤1.3), atrial (≥1.8), and pacemaker (1.4–1.7) cardiomyocyte subtypes (Figure 3c).^25^

**Figure 3:**
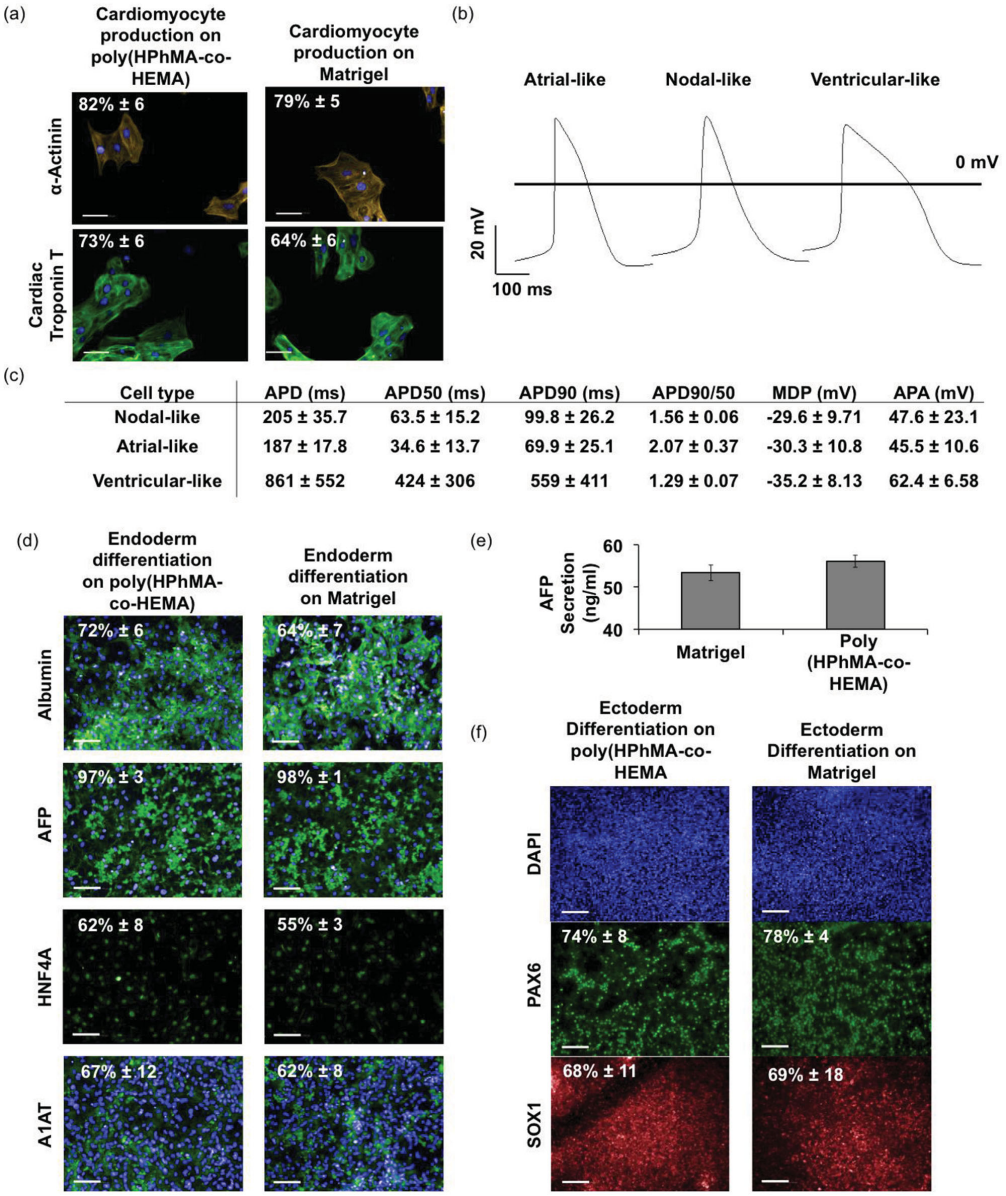
Three germ layer directed differentiation of hPSCs on polymeric substrate. a) Mesoderm differentiation on poly(HPhMA‐*co*‐HEMA) induced positive α‐actinin and cardiac troponin‐T expression of similar levels as cells induced on a Matrigel control surface. Scale bar = 50 μm. b) Electrophysiology of the spontaneously beating cardiomyocytes on poly(HPhMA‐*co*‐HEMA) showed all subtypes of cardiomyocytes, c) with a mean APD of 417 ± 102 ms. d) Endoderm differentiation on poly(HPhMA‐*co*‐HEMA) induced hepatic marker expression in hepatocyte‐like cells with positive endoderm expression (scale bar = 100 μm) and active AFP secretion e). f) Ectoderm differentiation on poly(HPhMA‐*co*‐HEMA) induced neurogenesis marker expression to similar levels as cells induced on a Matrigel control surface (scale bar = 100 μm).

Directed hepatocyte differentiation (endoderm) was achieved via an 18 d protocol using activin‐A, Wnt3a, FGF, HGF, and oncostatin‐M to modulate signaling cascades.^26^ Differentiated cell cultures on poly(HPhMA‐*co*‐HEMA)‐coated substrates expressed albumin, AFP, HNF4A, and A1AT and secreted AFP with comparable efficiency to Matrigel‐coated controls (Figure 3d,e).

Finally, we induced hPSC differentiation to neural progenitors, which arise from the ectoderm germ layer. Dual SMAD‐inhibition with dorsomorphin and SB431542 for 7 d induced the formation of neural rosette‐like colonies on poly(HPhMA‐*co*‐HEMA) substrates (Figure 3f).^27^, ^28^ Neural progenitors produced on Matrigel and poly(HPhMA‐*co*‐HEMA) displayed similar levels of PAX6 and SOX1 markers (PAX6: 78% ± 4% and 74% ± 8%; SOX1: 68% ± 11% and 69% ± 18%, respectively).

In summary, we have used a high throughput combinatorial approach to identify and develop a defined, synthetic polymeric substrate that supports hPSC pluripotency and expansion through serial passage in commercial defined media without the need for protein pre‐adsorption. This was achieved for both hESCs and hiPSCs. Additionally, directed differentiation was achieved on the hit polymer, poly(HPhMA‐*co*‐HEMA), to representatives of each of the three germ layers, including spontaneous beating clusters of cardiomyocytes (mesoderm), hepatocyte‐like cells (endoderm), and neuro‐ectoderm (ectoderm). It is proposed that the compatibility of this substrate with pluripotent cell expansion is consistent with the ready differentiation of these cells under the influence of soluble factors. Thus, poly(HPhMA‐*co*‐HEMA) fulfills all the current culture requirements for the clinical use of stem cells within regenerative medicine and can be scaled up by coating onto cultureware to be used off‐the‐shelf, providing a cost‐effective alternative to commercially available hPSC expansion substrates. The expansion of hPSCs and production of terminally differentiated cell types without the influence of undefined and xenogenic matrix protein coatings provides a robust platform for the industrial scale production of hPSCs for regenerative medicine applications and therapies.

## Experimental Section

*Preparation of Polymers*: polyHPhMA and poly(HPhMA‐*co*‐HEMA) were prepared via a thermally initiated free radical polymerization in an ethanolic solution with the addition of 2,2′‐azobis(2‐methylpropionitrile) (AIBN—1% w/w to HPhMA). The isolated and dried polymers were dissolved in ethanol (5% w/v) and added into TCPS six‐well to cover the base of each well plate directly after oxygen plasma activation. The solvent was allowed to evaporate under ambient conditions for 24 h prior to hPSC culture. Complete detailed methodology of polymer synthesis, characterization, and all cell culture protocols can be found in the Supporting Information.

## Supporting information

As a service to our authors and readers, this journal provides supporting information supplied by the authors. Such materials are peer reviewed and may be re‐organized for online delivery, but are not copy‐edited or typeset. Technical support issues arising from supporting information (other than missing files) should be addressed to the authors.

Supplementary

Supplementary
