# Supplementary material for: Discovery of a Novel Polymer for Human Pluripotent Stem Cell Expansion and Multilineage Differentiation
Source: Adv Mater. 2015 Jun 1;27(27):4006–12. doi: 10.1002/adma.201501351 (PMC4862031; doi:10.1002/adma.201501351)
Supplement: Supplementary file 1 — Supplementary [file ADMA-27-4006-s001.pdf]

Copyright WILEY-VCH Verlag GmbH & Co. KGaA, 69469 Weinheim, Germany, 2015.

ADVANCED MATERIALS

Supporting Information

for *Adv. Mater.,* DOI: 10.1002/adma.201501351

Discovery of a Novel Polymer for Human Pluripotent Stem Cell Expansion and Multilineage Differentiation

Adam D. Celiz, James G. W. Smith, Asha K. Patel, Andrew L. Hook, Divya Rajamohan, Vinoj T. George, Luke Flatt, Minal J. Patel, Vidana C. Epa, Taranjit Singh, Robert Langer, Daniel G. Anderson, Nicholas D. Allen, David C. Hay, David A. Winkler, David A. Barrett, Martyn C. Davies, Lorraine E. Young, Chris Denning, and Morgan R. Alexander**

Supporting Information

Discovery of a Novel Polymer for Human Pluripotent Stem Cell Expansion and Multi-Lineage Differentiation

**Adam D. Celiz, *James G. W. Smith, Asha K. Patel, Andrew L. Hook, Divja Rajamohan, Vinoj T. George, Minal J. Patel, Vidana C. Epa, Taranjit Singh, Robert Langer, Daniel G. Anderson, Nicholas D. Allen, David C. Hay, David A. Winkler, David A. Barrett, Martyn C. Davies, Lorraine E. Young, ★Chris Denning, ★Morgan R. Alexander.*

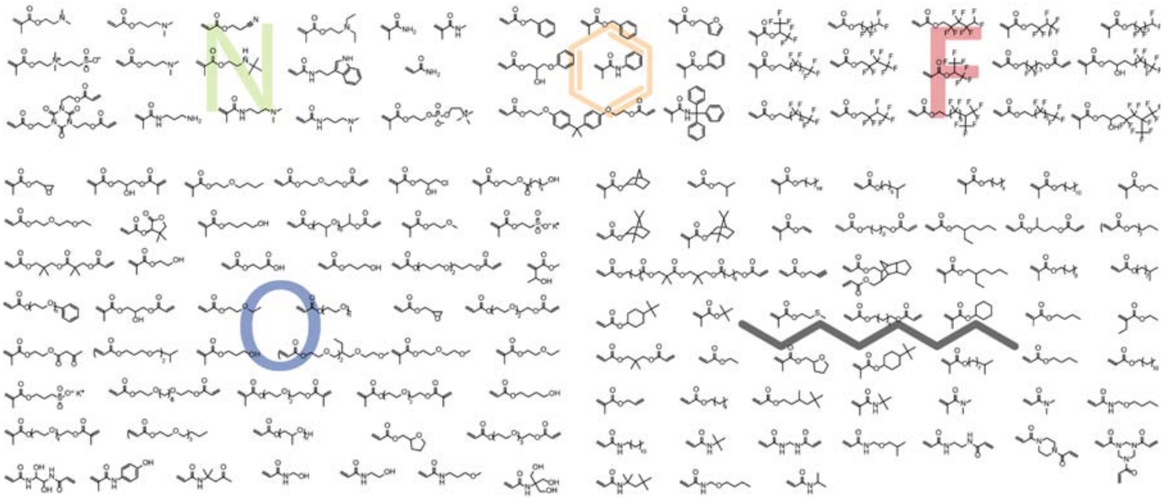

Supplementary Figure 1. Monomer structures of 141 materials used to form the first-generation array.

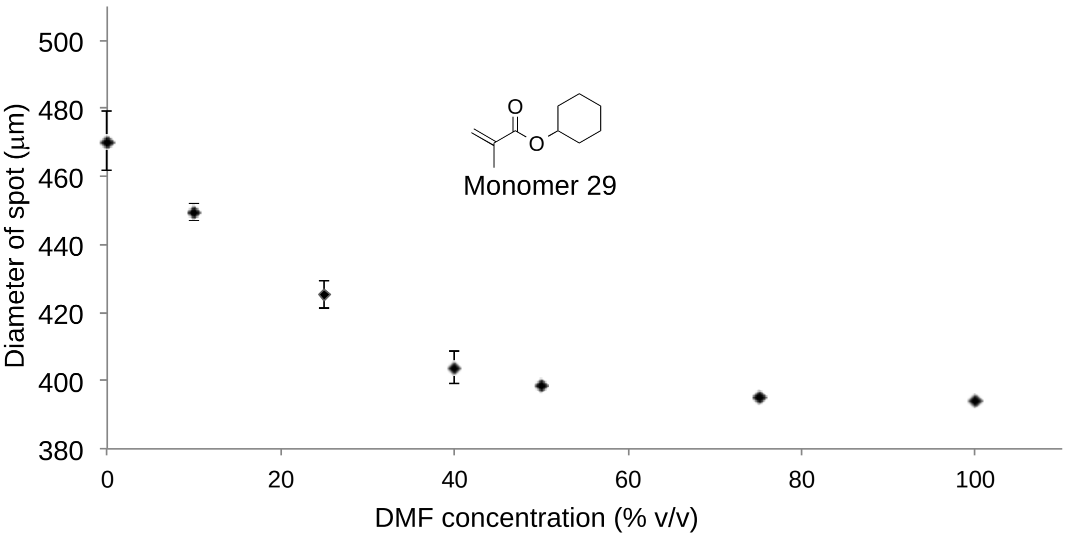

Supplementary Figure 2 | Spot diameters of monomer 29 in varying concentrations of DMF. A concentration of 50 % v/v was determined to be optimal for microarray printing.

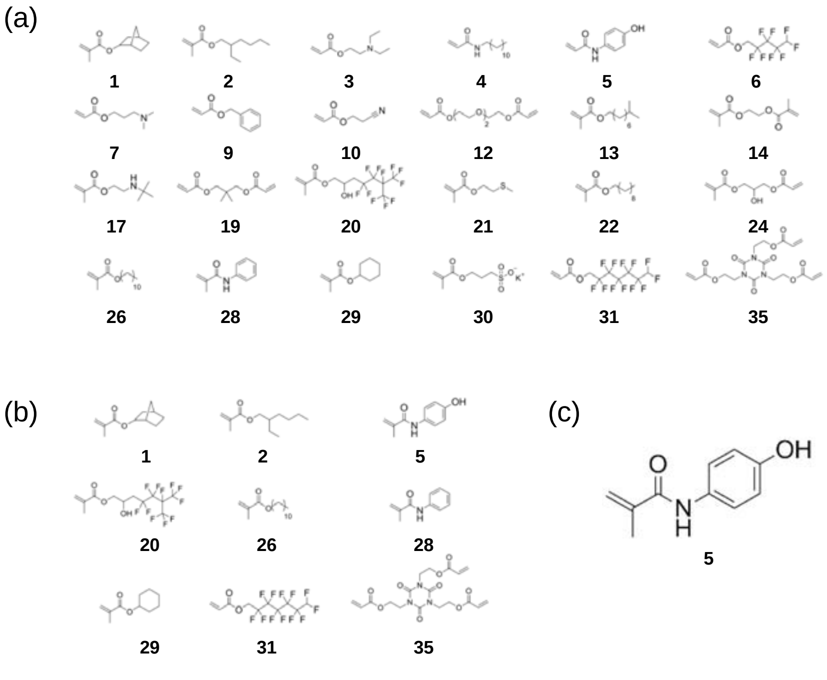

Supplementary Figure 3. Monomer structures of 'hit' materials identified from the (a) first-, (b) second- and (c) third-generation microarray.

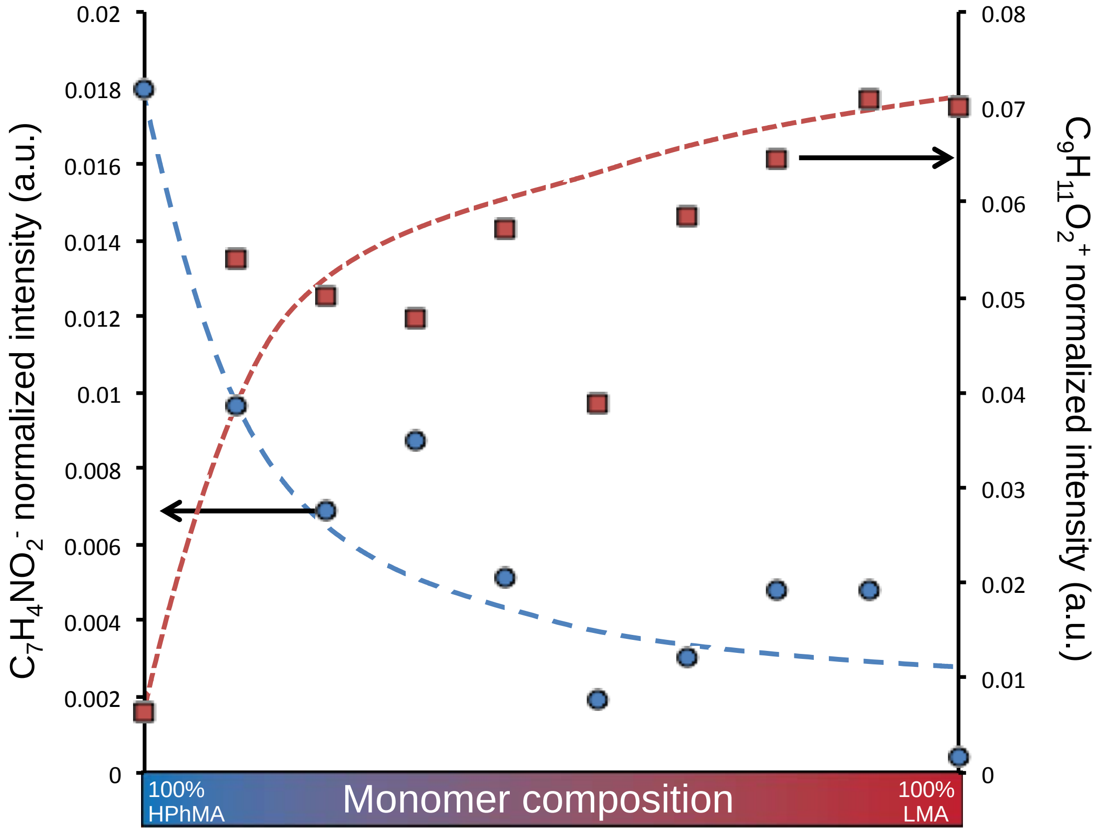

Supplementary Figure 4. ToF-SIMS ions characteristic to HPhMA ($C_7H_4NO_2^-$) and LMA ($C_9H_{11}O_2^+$) were compared in the spectra of the homopolymers and copolymers which revealed surface enrichment of M26 in poly(HPhMA-co-LMA) copolymers. Lines are drawn to guide the eye.

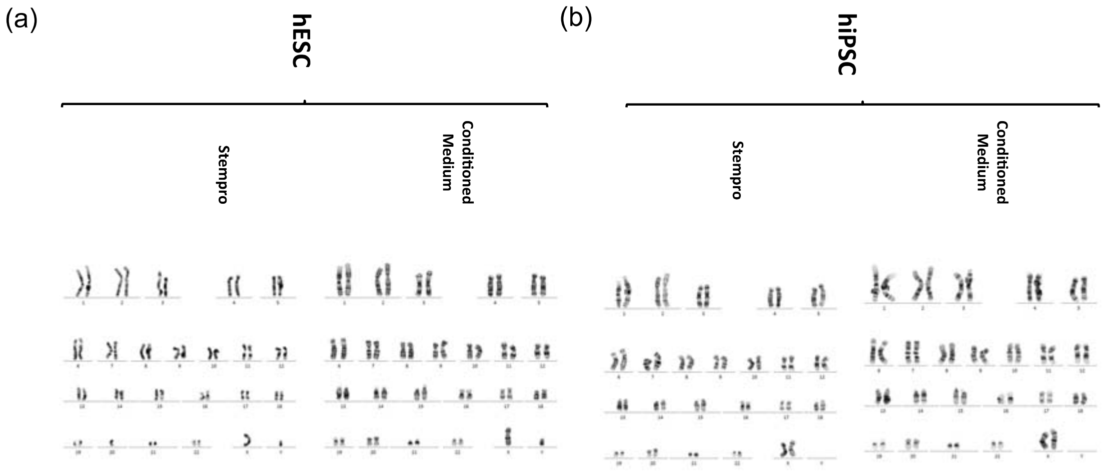

Supplementary Figure 5. Karyograms for (a) hESC (HUES7) and (b) hiPSC (BT1). Normal karyotype were observed after 5 serial passages (46,XY for HUES7; 46,XX for BT1) in StemPro and conditioned medium representative for 30 cells.

Supplementary methods

Polymer microarray synthesis. Polymer microarrays were formed using a XYZ3200 dispensing station (Biodot) and metal pins (946MP3B, Arrayit). The printing conditions were O_2 < 2000 ppm, 25 °C, and 35% humidity. Diluted polymerization solution was composed of monomer (50% v/v) in dimethylformamide with photoinitiator 2,2-dimethoxy-2-phenyl acetophenone (1% w/v). This monomer concentration was determined to optimal for printing and minimized spreading prior to photopolymerization (Supplementary Fig. 3). Six replicates were printed on each slide. Monomers were purchased from Aldrich, Scientific Polymers and Polysciences and printed onto epoxy-coated slides (Xenopore) dip-coated with polyHEMA (4% w/v, Sigma) in ethanol (95% v/v in water).

Microarray screening. The hESC line HUES7, was cultured on a Matrigel (BD Biosciences, UK) coated substrate in StemPro® medium containing 10 µm Y-27632 (Tocris Bioscience, UK), for no higher than passage 35, prior to seeding on arrays. Passaging of cells was achieved by incubation with accutase (Invitrogen, UK) for 3 minutes at 37 °C, with tapping of the flasks to dissociate cells. 1.2 x 10^6 HUES7 cells were seeded on each array and incubated at 37°C with 5% CO_2 for 24 hours to allow cell adhesion. Adherent cells were fixed in 4% paraformaldehyde (Sigma-Aldrich, UK) and permeabilized with 0.1% Triton-X 100 (Sigma-Aldrich, UK). Non-specific binding was blocked with 8% goat serum (Sigma-Aldrich, UK,) for 1 hour at room temperature. Samples were incubated with diluted mouse primary OCT4 antibody (1:200; Santa Cruz

Biotech, Germany) overnight at room temperature. Cy3-conjugated goat anti-mouse IgG+IgM secondary antibody (1:250; Jackson Immuno Research, Inc., PA) was applied for 1 hour at room temperature. Samples were incubated with 4',6-diamidino-2-phenylindole (DAPI) (1:1000; Sigma-Aldrich, UK,) for 10 minutes at room temperature and then mounted in Vectorshield mounting medium (Vector Labs, UK). Arrays were imaged using a fluorescence microscope (IMSTAR) and cell attachment determined using CellProfiler cell image analysis software (http://www.cellprofiler.org/).

Time-of-flight secondary-ion mass spectrometry. Measurements were conducted using a ToF-SIMS 4 (IONTOF GmbH) instrument operated using settings previously described[1].

X-ray photoelectron spectroscopy. XPS of microarrays was carried out on a Theta Probe (Thermo Scientific, East Grinstead, UK), which utilizes a micro-focused AlKα X-ray source (1486.6eV). The spot size for analysis was 100 μm and X-ray power was 20 W (15 kV, 1.33 mA). Dual ion charge neutralization was used throughout the analysis. The Theta Probe spectrometer was operated in standard lens mode (i.e. not angle-resolved). Survey spectra were collected at a pass energy of 200 eV, and were the average of 3 scans (dwell time 50 ms, step size 1 eV). High resolution spectra were collected at a pass energy of 40 eV and were the average of 10 scans (dwell time 100 ms, step size 0.1 eV). XPS of scaled up samples was carried out on a Kratos Axis Ultra instrument using monochromated Al Kα radiation (1486.6 eV), 15 mA emission current, 10 kV anode potential and a charge-compensating electron flood. High-resolution core levels were acquired at a pass energy of 20 eV.

Preparation of polymers. HPhMA was polymerized in an ethanolic solution via a thermally initiated free radical polymerization with the addition of 2,2'-azobis(2-methylpropionitrile) (AIBN – 1% w/w to HPhMA). The isolated and dried polymer (Yield = 51%, M_w: 91 kDa, PDI: 1.41 - Figure S8) was dissolved into ethanol (5% w/v) and added into TCPS 6-well to cover the base of each well plate directly after oxygen plasma activation. The solvent was allowed to evaporate under ambient conditions for 24 hours prior to hPSC culture.

Preparation of polymer-coated cultureware. TCPS six-well plates were oxygen plasma etched in a Si-free reactor at 100 W for 10 minutes prior to coating with polymer solution. Prepolymerised polymer was dissolved in ethanol to make a 5% w/v solution. 100 μL of polymer solution was pipetted into each well, the plate was loosely closed and allowed to dry overnight at ambient conditions.

Microarray screening. Arrays were sterilized and cultured with hPSCs as described previously[1]. Initial seeding densities of HUES7 cells on first-, second- and third-generation arrays were 1 x 10^6, 1 x10^6 and 8 x 10^5 cells respectively.

Expansion culture on scaled up surfaces. Top and bottom surfaces of coated 6 well plates were sterilized by exposure to UV light for 15 minutes each, followed by washing with sterile PBS three times. 6 x 10^5 hPSCs (HUES7 or BT1) were seeded per well in StemPro® medium containing 10 μm Y-27632 dihydrochloride. Medium was exchanged every 24 hours until cells reached confluence. After 72 hours cell passaging was achieved by incubation with accutase (Invitrogen, UK) for 3 min at 37 °C, with tapping of the flasks to dissociate cells.

Ectoderm differentiation protocol. To induce ectoderm differentiation 2 x 10^4 hPSCs were seeded per well of poly(HPhMA-co-HEMA)-coated 96 well plates. Following 24 hours adhesion, cells were subjected to daily media exchanges for seven days with 100 µl ectoderm inducing medium that comprised of advanced DMEM base medium (Life Technologies), supplemented with 1 x L-glutamine (Life Technologies), 1 x CD Lipid Concentrate (Life Technologies), 7.5 µg/ml Transferrin (Sigma Aldrich), 14 µg/ml Insulin (Sigma Aldrich), 0.1mM β-mercapto-ethanol, 10 µM SB431542 (Tocris), and 1 µM Dorsomorphin-1 (Tocris).

Endoderm differentiation protocol. 1.7x10^4 hPSCs were seed per well of poly(HPhMA-co-HEMA)-coated 96 well plates. Following 24 hours adhesion cells were subjected to a protocol described elsewhere.[2]

Mesoderm differentiation protocol. Differentiation into mesoderm was adapted from the original monolayer protocol for production of cardiomyocytes[3]. Briefly, 2.5 x 10^4 undifferentiated hPSCs were seeded per well of polymer poly(HPhMA-co-HEMA) coated 96 well plates and allowed to expand for 72 hours until confluent. At this stage, (day 1 of differentiation) cells per well were subjected to 100 µl mesoderm-inducing medium A consisting of StemPro-34 (Life Technologies) medium containing growth factors, recombinant human Activin A (Life Technologies; 10-15 ng/ml) and recombinant human BMP4 (R&D Systems, 10 ng/ml). Media exchange was performed

on day 3 with 100 µl mesoderm inducing medium B- consisting of RPMI base medium (Life Technologies) supplemented with 1 x B-27 (Life Technologies) and a small molecule inhibitor, KY02111 (R&D Systems). From day 5 onwards, cells were maintained in RPMI base medium supplemented with B-27 only, with media changes every 2-3 days.

Patch Clamp. Whole cell recordings were performed in the current clamp mode using an ECP-10 HEKA amplifier. Cells were maintained in NT Buffer (140 mM NaCl, 10 mM glucose, 10 mM HEPES, 4 mM KCl, 1 mM $MgCl_2$, 1.8 mM $CaCl_2$, pH 7.45/NaOH) and at near-physiological temperatures (37±2 °C). Patch pipettes were pulled on a Sutter P-97 programmable micropipette puller and had resistances of between 2 and 5 MΩ when filled with the internal solution (145 mM KCl, 5 mM NaCl, 2 mM $CaCl_2$, 2 mM $MgCl_2$, 4 mM EGTA, 10 mM HEPES, pH 7.3/KOH). The softwares Pulse (HEKA) and Clampfit v9.0 (Molecular Devices) were used for data acquisition and analysis respectively.

Immunocytochemistry staining and quantification. Cell cultures were fixed in 4% paraformaldehyde (Sigma-Aldrich, UK) and permeabilized with 0.1% Triton-X 100 (Sigma-Aldrich, UK). Non-specific binding was blocked with 8% goat serum (Sigma-Aldrich) for 1 hour at room temperature. Samples were incubated with diluted primary antibody (Table S3) overnight at room temperature. Species secondary antibody (Table S3) was then applied for 1 hour at room temperature. Samples were counter-stained with 4',6-diamidino-2-phenylindole (DAPI) (1:1000; Sigma-Aldrich, UK,). High content

analysis was performed to determine the percentage positive cells within cultures using an Operetta® confocal plate reader (Perkin-Elmer).

References

[1] A. D. Celiz, J. G. W. Smith, A. K. Patel, R. Langer, D. G. Anderson, D. A. Barrett, L. E. Young, M. C. Davies, C. Denning, M. R. Alexander, *Biomater. Sci.* **2014**, *2*, 1604.

[2] D. C. Hay, S. Pernagallo, J. J. Diaz-Mochon, C. N. Medine, S. Greenhough, Z. Hannoun, J. Schrader, J. R. Black, J. Fletcher, D. Dalgetty, A. I. Thompson, P. N. Newsome, S. J. Forbes, J. A. Ross, M. Bradley, J. P. Iredale, *Stem Cell Res.* **2011**, *6*, 92.

[3] C. Xu, S. Police, M. Hassanipour, Y. Li, Y. Chen, C. Priest, C. O'Sullivan, M. A. Laflamme, W.-Z. Zhu, B. Van Biber, L. Hegerova, J. Yang, K. Delavan-Boorsma, A. Davies, J. Lebkowski, J. D. Gold, *Regen. Med.* **2011**, *6*, 53.
